# Network analyses elucidate the role of SMYD3 in esophageal squamous cell carcinoma

**DOI:** 10.1002/2211-5463.12251

**Published:** 2017-07-03

**Authors:** Xinning Liu, Zhoude Zheng, Chuhong Chen, Simin Guo, Zhennan Liao, Yue Li, Ying Zhu, Haiying Zou, Jianyi Wu, Wenming Xie, Pixian Zhang, Liyan Xu, Bingli Wu, Enmin Li

**Affiliations:** ^1^ Key Laboratory of Molecular Biology in High Cancer Incidence Coastal Chaoshan Area of Guangdong Higher Education Institutes Shantou University Medical College China; ^2^ Department of Biochemistry and Molecular Biology Shantou University Medical College China; ^3^ Network and Information Center Shantou University Medical College China; ^4^ Institute of Oncologic Pathology Shantou University Medical College China

**Keywords:** functional enrichment annotation, protein–protein interaction network, SMYD3

## Abstract

SMYD3 is a member of the SET and myeloid‐Nervy‐DEAF‐1 (MYND) domain‐containing protein family of methyltransferases, which are known to play critical roles in carcinogenesis. Expression of SMYD3 is elevated in various cancers, including esophageal squamous cell carcinoma (ESCC), and is correlated with the survival time of patients with ESCC. Here, we dissect gene expression data, from a previously described KYSE150 ESCC cell line in which SMYD3 had been knocked down, by integration with the protein–protein interaction (PPI) network, to find the new potential biological roles of SMYD3 and subsequent target genes. By construction of a specific PPI network, differentially expressed genes (DEGs), following SMYD3 knockdown, were identified as interacting with thousands of neighboring proteins. Enrichment analyses from the DAVID Functional Annotation Chart found significant Gene Ontology (GO) terms associated with transcription activities, which were closely related to SMYD3 function. For example, YAP1 and GATA3 might be a target gene for SMYD3 to regulate transcription. Enrichment annotation of the total DEG PPI network by GO ‘Biological Process’ generated a connected functional map and found 532 significant terms, including known and potential biological roles of SMYD3 protein, such as expression regulation, signal transduction, cell cycle, cell metastasis, and invasion. Subcellular localization analyses found that DEGs and their interacting proteins were distributed in multiple layers, which might reflect the intricate biological processes at the spatial level. Our analysis of the PPI network has provided important clues for future detection of the biological roles and mechanisms, as well as the target genes of SMYD3.

**Abbreviations**
DEGs: differentially expressed genes
GO: Gene Ontology
PPI: protein–protein interaction
SMYD3: SET and MYND domain‐containing 3

Methylation of histone proteins plays a pivotal role in the regulation of a wide range of biological processes. SET and myeloid‐Nervy‐DEAF‐1 (MYND) domain‐containing protein (SMYD) is a methyltransferase family that includes SMYD1, SMYD2, SMYD3, SMYD4, and SMYD5 and has been found to play critical roles in human carcinogenesis. Altered expression of SMYD3 is associated with the progression of several solid tumors, including bladder cancer 1, glioma 2, gastric cancer 3, and prostate cancer 4. Sarris *et al*. 5 found a significant correlation between elevated expression of SMYD3 and the incidence of both hepatocellular carcinoma and colorectal cancer. Several trials have explored the effects of SMYD3 overexpression on proliferation, viability, cancer cell migration, and invasion 6, 7, 8, 9, 10. The series of elegant experiments suggested that SMYD3 could serve as a potential biomarker for clinically aggressive disease and an attractive therapeutic target. The biological roles for SMYD3 are numerous and far beyond its transactivation activity. Thus, more attention directed toward other roles remains necessary.

Network‐based analysis of protein–protein interactions (PPI) refers to the association among protein molecules and the study of these associations from the aspects of biochemistry, signal transduction, and biomolecular networks. Proteins do not work alone, interacting with other proteins in the biological context of specific functions 11. In recent years, integrated analysis of large‐scale gene expression data, or other high‐throughput data, with the PPI network has received great attention to elucidate biological processes 12. Integration analyses with the PPI network provide a number of applications, such as protein interaction prediction, disease candidate genes identification, protein function prediction, functional protein module identification, protein complex, and drug target identification 13.

In our previous study, we found that SMYD3 expression is frequently upregulated in human esophageal squamous cell carcinoma (ESCC) tissues, correlating with overall survival of patients with ESCC 14. RNAi‐mediated knockdown of SMYD3 suppressed ESCC cell proliferation, migration, and invasion *in vitro* and inhibited local tumor invasion *in vivo* 14. Future analyses should be carried out beyond the mere listing of differentially expressed genes (DEGs) after microarray experiments or other high‐throughput experiments. It would be helpful to explain the biological roles or biological phenotype of target genes, especially when more and more spatial or temporal interactions between proteins are obtained from the public databases. In this study, we analyzed DEGs, following SMYD3 knockdown in ESCC cells, by applying PPI network analysis.

## Materials and methods

### Expression of SMYD3 in ESCC from TCGA data

Expression data of esophageal carcinoma (TCGA_ESCA_exp_HiSeq‐2015‐02‐24) were downloaded from TCGA (https://cancergenome.nih.gov/), which contained level 3 expression of 89 cases of ESCC by RNA‐seq. The X‐tile program 3.6.1 15, to define the optimal cutoff point for the expression level for SMYD3, was used to classify patients with ESCC into two groups, high and low SMYD3 expression, following the Kaplan–Meier survival analysis and log‐rank test by SPSS 13.0 software (version 13.0; SPSS, Inc., Chicago, IL, USA).

### The expression profile and differentially expressed genes

SMYD3 was knocked down as described in our previous study 14. Briefly, shRNA sequences targeting SMYD3 were ligated into the pGLV3/H1/GFP/+ Puro vector and transfected into the KYSE150 ESCC cell line. KYSE150 cells were transfected with an empty plasmid as a control. SMYD3 knockdown was confirmed by both QRT‐PCR and western blot, and the mRNA expression profile was analyzed with the GeneChip® PrimeView™ Human Gene Expression Array (Affymetrix, Santa Clara, CA, USA). Raw data were treated by normalization and log transformation. Both raw and treated expression data have been submitted to the NCBI GEO database (http://www.ncbi.nlm.nih.gov/geo/) and were assigned as GSE85419. A threshold of twofold change was set for DEGs in this study.

### Construction of the PPI network

Currently known human PPI data are available from the newest releases of the following databases: HPRD (http://www.hprd.org/), BioGRID (http://thebiogrid.org/), DIP (http://dip.doe-mbi.ucla.edu/dip/Main.cgi), and IntAct (http://www.ebi.ac.uk/intact/). These physical protein interactions were collected from public reports of both low‐throughput and high‐throughput experimental results, providing high confidence for the following analyses, such as disease researches integrated with the human PPI network 16, 17.

At first, we manually integrated the data to obtain a unique dataset of interactions for the Homo sapiens species. These unique PPI data were considered as a curated parental PPI network, containing 18 644 unique proteins and 199 411 interactions, which was applied for new or child PPI network construction. Cytoscape software has been widely applied for visualization, data integration, and analyses of PPI networks, as it provides update plugins to meet the needs of large‐scale data analyses 18. In Cytoscape, for visualization as graphs, PPI networks are presented with nodes as proteins and the edges as their interactions. First, the total DEGs, downregulated DEGs, and upregulated DEGs were mapped to the parental PPI network, and extracted their first class interacting neighbors to construct three PPI subnetworks. To increase the reliability and reduce the unnecessary connections, the network reconstruction was limited to the first interacting protein neighbors of these DEGs. Second, to detect the axis of SMYD3‐neighboring proteins, SMYD3 was used as the query node to construct a SMYD3‐central PPI network. Third, a subnetwork was created to detect the internal interactions between DEGs by mapping all DEGs to the parental PPI network.

### Analyses of PPI network topological parameters

The analyses of multiple topological parameters of networks were carried out by NetworkAnalyzer 19. The network topological parameters are important characters to understand the organization of complex networks, such as the PPI network 20. The degree is defined as the number of one node's directly interacting protein neighbors in the PPI network. One of most important network topological characteristics is that the degree distribution follows a scale‐free power law distribution for many large networks, such as the PPI network or social network. In this study, the power law of distribution of node degrees was analyzed by the method in our previous report 21.

The three new PPI subnetworks were treated as undirected in this study. The degree distribution *P*(*k*) of a large‐scale network is defined as the fraction of nodes in the network with degree *k*. The pattern of their dependencies can be visualized by fitting a line on the node degree distribution data. NetworkAnalyzer calculates the positive coordinate value for fitting the line where the power law curve of the form *y* = β*x* ^*a*^. *R* ^2^ value is a statistical measure of the linearity of the curve fit and used to quantify the fit to the power line. When the fit is good, the *R* ^2^ value is very close to 1. Moreover, many topological parameters were also analyzed and shown, as they indicate the network properties.

### Gene functional enrichment analyses

The Functional Annotation Chart in DAVID (http://david.abcc.ncifcrf.gov/) is able to examine the significance of gene‐term enrichment by the application of a modified Fisher's exact test 22. The chart covers 40 annotation categories, including GO terms, protein functional domains, pathways, sequence features, disease associations, homology, gene functional summaries, and literature. Terms from the Functional Annotation Chart with *P* < 0.05 were visualized by Enrichment Map 23, which organizes the enriched overlapped gene sets into a network.

### Generation of the functional annotation map

The total DEG PPI network was annotated by Gene Ontology (GO) for the mining of enriched GO ‘Biological Process’ terms using the ClueGO plugin, which creates a functionally and hierarchical organized GO/pathway term network 24. The enriched annotated terms with a *P*‐value < 0.001 were defined as significant. To visualize the relationship between GO terms, a kappa score, reflecting the overlapping number between terms, was set to 0.3 as the threshold.

### Subcellular classes of the total DEG PPI network

The subcellular localization for proteins in the total DEG PPI network was obtained from the ‘GENE‐ONTOLOGY annotation file’ in the HPRD database, which was curated and imported into the network as a node attribute. For the localizations of several proteins that were annotated by multiple locations, including proteins that might translocate into the nucleus (e.g., both in cytoplasm and in nucleus), these localizations were merged (e.g., cytoplasm/nucleus). Cerebral program is able to separate the nodes in the total DEG PPI network into multiple layers according to their subcellular localization remaining their interactions, generating a pathway‐like graph 25.

## Results

### Correlation of SMYD3 with survival of patients with ESCC

**Figure 1:**
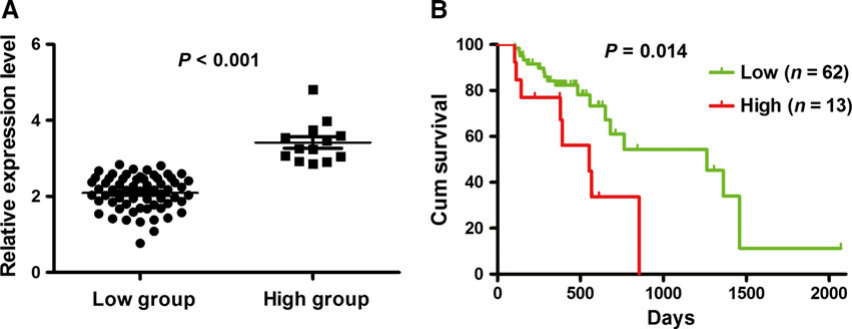
Expression of SMYD3 correlates with survival of patients with ESCC. (A) The significant difference between low and high expression level of SMYD3. (B) The lower expression level of SYMD3 favors a long survival time for patients with ESCC.

Although we previously found that SMYD3 is overexpressed in patients with ESCC from China, we applied another dataset as a validation cohort. By X‐tile, patients with ESCC were classified into two groups according to the expression level of SMYD3 (*P* < 0.001; Fig. 1A). Compared with patients with high SYMD3 expression, ESCC patients with lower SYMD3 had a longer survival time (*P* = 0.014), consistent with our previous report (Fig. 1B).

### The construction of three DEG PPI networks

Using twofold as the threshold, we identified 238 DEGs (85 upregulated genes and 153 downregulated genes) from the mRNA expression microarray of SMYD3‐knockdown ESCC cells. In order to provide a landscape of what and how the DEGs participate in cellular biological activities, we constructed a full view of their interaction proteins to shed light on their functions, as well as the biological role of SMYD3 in ESCC. The PPI datasets from several acknowledged interaction databases collected from the literature provide original and credible data for performing research. By mapping the total, downregulated, and upregulated DEGs to the parental PPI network and extracting their first interacting neighbors, three sub‐PPI networks were generated.

**Figure 2:**
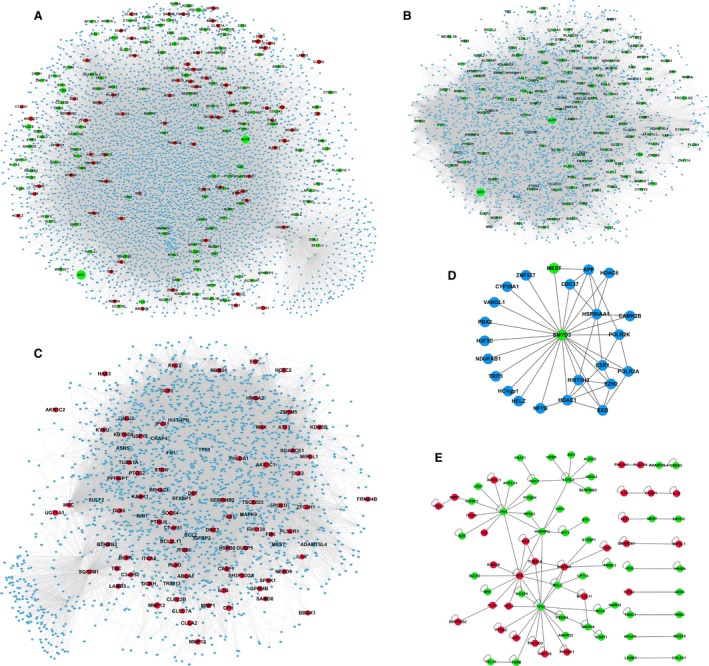
PPI network generation by mapping DEGs to the parental PPI network. (A) PPI network of total DEGs. (B) PPI network of downregulated DEGs. (C) PPI network of upregulated DEGs. (D) SMYD3‐central PPI subnetwork. (E) Internal interactions of DEGs. Different node colors indicate the types of proteins represented. Green and red nodes represent proteins encoded by down‐ and upregulated genes, respectively. Blue nodes represent interacting proteins that were not significantly differentially expressed.

As shown in Fig. 2A, the total DEG PPI subnetwork contained 4426 nodes and 82 039 edges, including 204 DEGs. The literature reports that 129 downregulated DEGs have interacting proteins to form a sub‐PPI network composed of 2963 nodes and 58 833 edges (Fig. 2B). Third, 85 upregulated proteins formed a PPI subnetwork containing 2176 nodes and 37 238 edges (Fig. 2C). These three PPI subnetworks suggested that the knockdown of SMYD3 tremendously disturbs the protein activities in ESCC, as more than 200 DEGs, resulting from SMYD3 knockdown, were capable of broadly influencing biological processes through the interactions with thousands of other proteins.

Currently, 23 SMYD3‐interacting proteins have been identified. Nevertheless, the expression levels of these 23 proteins were not significantly changed, except for the gene mesoderm‐specific transcript (MEST), which was downregulated in the mRNA profile of SMYD3 knockdown in ESCC (Fig. 2D). The DEG–DEG interactions were acquired from the parental PPI network to detect their internal interactions. The DEG–DEG network contained 77 nodes (48 downregulated and eight upregulated DEGs) and 120 edges, which also included a large module containing 59 DEGs (39 downregulations and 20 upregulations; Fig. 2E).

### Network topological characteristics analyses

**Figure 3:**
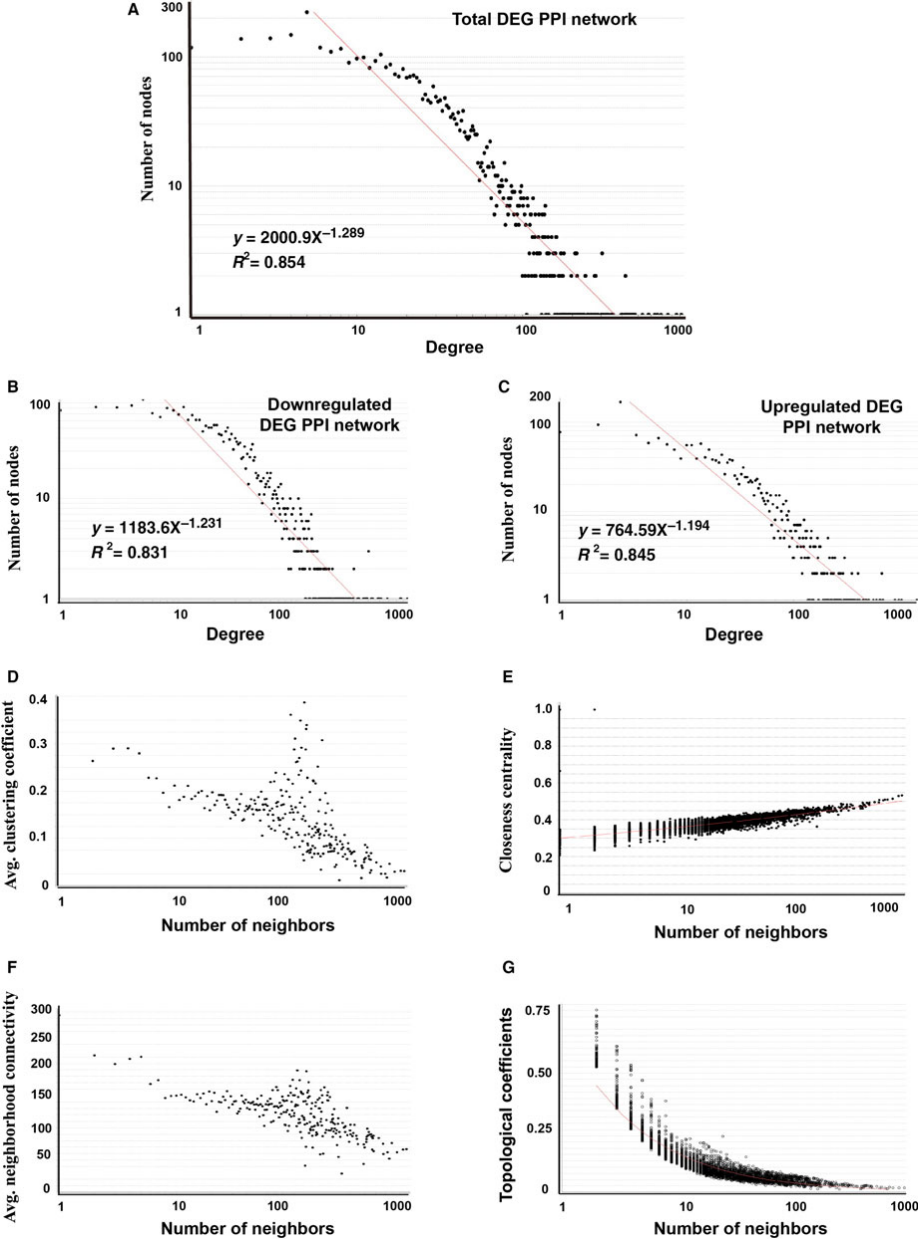
Power law distribution of node degree of the total DEG PPI network (A), downregulated DEG PPI network (B), and upregulated DEG PPI subnetwork (C). The graph displays a decreasing trend of degree distribution with an increase in number of links displaying scale‐free topology. The results of average clustering coefficient distribution (D), closeness centrality (E), neighborhood connectivity distribution (F), and topological coefficients (G) were shown.

Based on the specific distinguishing principles (e.g., power law distribution of node degree), a real biological network, such as the PPI network, is significantly discriminated from random networks 26, 27. For the total, downregulated, and upregulated DEG networks, the node degree distributions approximately showed a power law distribution, with an *R* ^2^ = 0.854, 0.831, and 0.845, respectively (Fig. 3A–C). These results indicated the three DEG PPI subnetworks constructed in this study were real complex biological networks characterized scale‐free 28. It also suggested that a small number of important proteins act as hub nodes with a large amount of interactions. Three topological metrics proposed to understand the structure of a complex network, specifically network density, network centralization, and clustering coefficient, are shown in Table 1. Several other important network characteristics, for example, average clustering coefficient distribution, closeness centrality, neighborhood connectivity distribution, and topological coefficients, are indicated in Fig. 3D–G.

**Table 1:** Topological parameters of three DEGs PPI subnetwork

PPI subnetwork	*y* = β*x* ^*a*^	*R* ^2^	Correlation	Clustering coefficient	Network centralization	Network density
Total DEGs	*y* = 2000.9*x* ^**−**1.289^	0.854	0.514	0.188	0.206	0.009
Downregulated DEGs	*y* = 1183.6*x* ^**−**1.231^	0.831	0.511	0.200	0.275	0.013
Upregulated DEGs	*y* = 764.59*x* ^−1.194^	0.814	0.619	0.240	0.254	0.013

### Functional annotation map of DEGs

**Figure 4:**
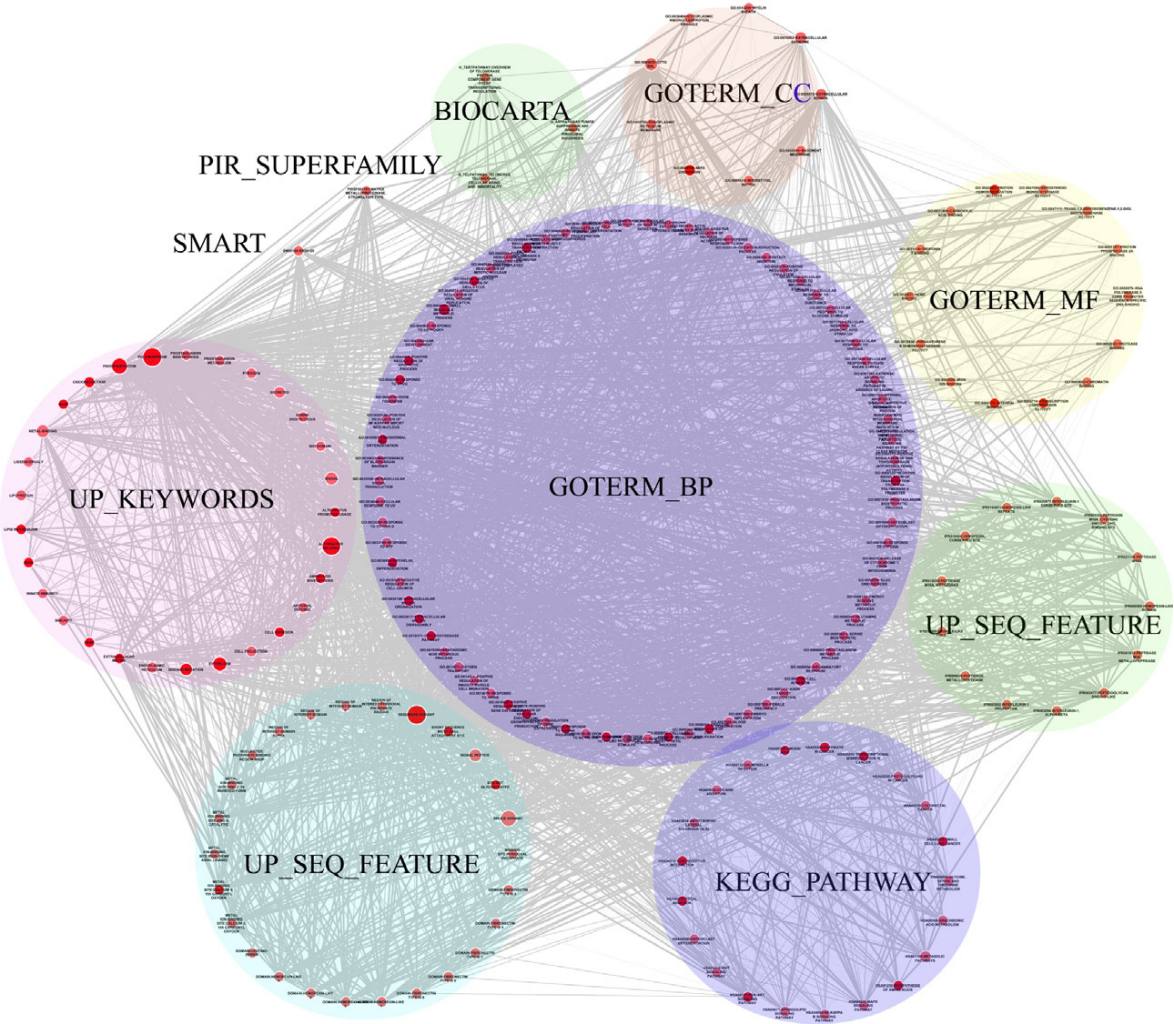
Enrichment map for the DEGs to identify significant biological functions (*P* < 0.05). One node represented a significant functional term from the Functional Annotation chart. Node size represents enrichment significance. Edges indicate overlap between gene sets, whereas the thickness indicates the size of the overlap.

To gain a full view of the functions and categories of the DEGs, the DEGs were annotated by Functional Annotation Chart and visualized by Enrichment Map. As shown in Fig. 4, one node represents one functional annotation term. The more significant the enriched term, the larger it is. Nodes from the same kind of functional category are shown in the same color. The edge width, defined by the overlap coefficient between the enriched terms (overlap coefficient cutoff was set as 0.6), is wider when more of the same genes overlap in two nodes.

The Functional Annotation Chart results contained another 94 terms from the following annotation categories: 21 KEGG_PATHWAY, 13 INTERPRO, 26 UP_SEQ_FEATURE, 1 SMART, 29 SP_PIR_KEYWORDS, and 3 BIOCARTA (Appendix S1). These results would provide more information than mere GO enrichment. Accumulated evidence suggests that SMYD3 can influence distinct oncogenic processes by acting as a gene‐specific transcriptional regulator. This is supported by the DEGs from its knockdown being involved the transcription activities, such as two terms from GOTERM_BP_DIRECT: ‘GO:0010468~regulation of gene expression’ (PTHLH, BCL2, PHGDH, MYC, PHLDA2) and ‘GO:0010628~positive regulation of gene expression (PLSCR1, INHBA, EZR, TNC, TP53, MAPK9, IL1B, HMGA2, KDM5B, HNRNPU, IL1A, FN1)’. SMYD3 is also a histone lysine methyltransferase. Two significant terms about transcriptional regulation at chromosome level were found. GO:0003682 of ‘chromatin binding’ contained 10 enriched genes: DLX2, NUPR1, SMARCE1, GATA3, ANKRD2, TP53, BCL6, yes‐associated protein 1(YAP1), LOXL2, and PLAC8. GO:0000979 of ‘RNA polymerase II core promoter sequence‐specific DNA binding’ contained four enriched genes: GATA3, TP53, SMYD3, and YAP1. The term ‘domain: Leucine‐zipper’ from UP_SEQ_FEATURE contained five DEGs: MAX, TSC22D3, ATF3, CREB5, and MYC, indicating their protein sequence characters involved DNA binding in transcription regulation. These results might explain SMYD3 functioning as a histone methyltransferase, and why its knockdown in tumor cells extensively affects gene expression regulation activities.

### Generation of functional annotation map

**Figure 5:**
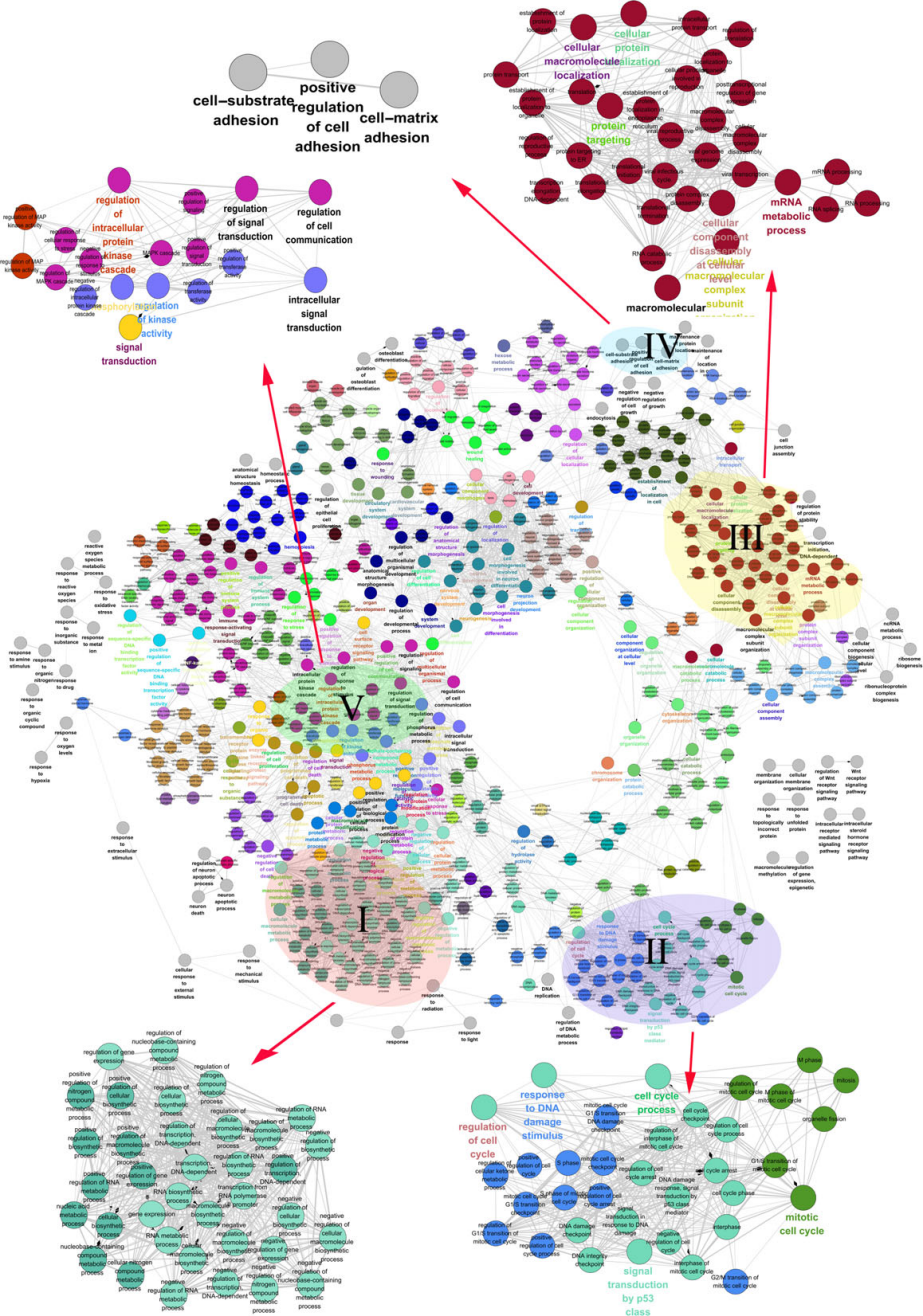
Functional map of the total DEG PPI network. Functionally grouped network with terms is linked as nodes based on their kappa score (≥ 0.3). Functional groups with overlapped enriched genes are linked by an edge. Similar GO terms are labeled in the same color. The interested GO term related or potentially related to known and potential functions of SMYD3 is grouped by the Roman numeral. I: gene expression regulation‐associated terms; II: cell cycle‐associated terms; III: protein synthesis and RNA processing; IV: cancer cell metastasis and invasion; V: signal regulation or signal transduction.

Cellular activities might be affected by the DEGs through cascades of interactions in the network to perform their multiple biological functions. To find potential cellular activities perturbed by SMYD3 protein through its DEGs and their interacting proteins, GO ‘Biological Process’ enrichment analyses of the total DEG PPI network were performed. We generated 532 enriched GO terms to construct a functional annotation map, in which the nodes were no longer proteins, but rather their enriched GO terms, with the edges suggesting significant overlapping of enriched proteins between two GO terms (Fig. 5).

Interestingly, many groups of GO terms possibly associated with SMYD3 functions were found, as indicated by capitalized Roman numerals. As SMYD3 plays important roles in histone modification, a group of gene expression regulation‐associated terms were identified, such as ‘gene expression’, ‘regulation of gene expression’, ‘positive regulation of transcription, DNA‐dependent’, and ‘transcription from RNA polymerase II promoter’. The second interesting result was that many of the proteins from the total DEG PPI subnetwork participated in signal regulation or signal transduction, for example, ‘signal transduction’, ‘intracellular signal transduction’, ‘regulation of cell communication’, ‘regulation of intracellular protein kinase cascade’. A large functional group contained several cell cycle‐associated GO terms, for example, ‘cell cycle process’, ‘mitotic cell cycle’, ‘regulation of cell cycle’, ‘cell cycle arrest’, and ‘respond to DNA damage stimulus’, suggesting that SMYD3 regulates the cell cycle directly or indirectly by the cascade of protein interaction. Three terms indicating the known biological roles of SMYD3 in promoting cancer cell metastasis and invasion were also found: ‘cell–substrate adhesion’, ‘cell–matrix adhesion’, and ‘positive regulation of cell adhesion’. The interesting significant important GO terms are listed in Table 2.

**Table 2:** Interesting significant GO terms for SMYD3‐knockdown PPI network

Significant GO list	Term name	*P*‐value corrected with Bonferroni
mRNA translation‐related terms		
GO:0043933	Macromolecular complex subunit organization	1.36E‐56
GO:0016071	mRNA metabolic process	9.51E‐50
GO:0070727	Cellular macromolecule localization	1.18E‐44
GO:0034613	Cellular protein localization	2.27E‐44
GO:0006886	Intracellular protein transport	4.69E‐42
GO:0033365	Protein localization to organelle	1.16E‐37
GO:0006412	Translation	7.26E‐35
GO:0006413	Translational initiation	1.16E‐32
GO:0034623	Cellular macromolecular complex disassembly	2.36E‐32
GO:0006414	Translational elongation	8.80E‐32
GO:0006415	Translational termination	9.32E‐32
GO:0045047	Protein targeting to ER	9.92E‐32
GO:0072599	Establishment of protein localization in endoplasmic reticulum	9.92E‐32
GO:0043241	Protein complex disassembly	1.00E‐27
GO:0015031	Protein transport	7.98E‐26
GO:0045184	Establishment of protein localization	9.67E‐26
GO:0048610	Cellular process involved in reproduction	1.07E‐24
GO:0006401	RNA catabolic process	1.94E‐24
GO:0010608	Post‐transcriptional regulation of gene expression	6.50E‐18
GO:0006396	RNA processing	1.08E‐17
GO:0072594	Establishment of protein localization to organelle	3.65E‐17
GO:0008380	RNA splicing	6.43E‐15
GO:0006397	mRNA processing	4.23E‐14
GO:2000241	Regulation of reproductive process	1.84E‐05
GO:0006417	Regulation of translation	2.13E‐05
Signal pathway‐related terms		
GO:0016310	Phosphorylation	5.89E‐51
GO:0035556	Intracellular signal transduction	1.15E‐38
GO:0009966	Regulation of signal transduction	1.34E‐37
GO:0010646	Regulation of cell communication	2.61E‐37
GO:0007165	Signal transduction	1.07E‐33
GO:0043549	Regulation of kinase activity	1.32E‐30
GO:0051338	Regulation of transferase activity	4.04E‐30
GO:0048585	Negative regulation of response to stimulus	9.24E‐24
GO:0000165	MAPK cascade	1.14E‐19
GO:0010627	Regulation of intracellular protein kinase cascade	1.31E‐19
GO:0080135	Regulation of cellular response to stress	2.68E‐19
GO:0051347	Positive regulation of transferase activity	2.49E‐18
GO:0009967	Positive regulation of signal transduction	6.47E‐17
GO:0023056	Positive regulation of signaling	1.56E‐15
GO:0043408	Regulation of MAPK cascade	6.18E‐13
GO:0043405	Regulation of MAP kinase activity	3.37E‐11
GO:0043406	Positive regulation of MAP kinase activity	2.86E‐07
GO:0010741	Negative regulation of intracellular protein kinase cascade	4.25E‐07
Cell cycle‐related terms		
GO:0051726	Regulation of cell cycle	5.94E‐48
GO:0022402	Cell cycle process	7.59E‐43
GO:0000278	Mitotic cell cycle	1.28E‐41
GO:0051329	Interphase of mitotic cell cycle	5.92E‐36
GO:0051329	Interphase of mitotic cell cycle	5.93E‐36
GO:0045786	Negative regulation of cell cycle	1.92E‐33
GO:0022403	Cell cycle phase	1.05E‐31
GO:0007050	Cell cycle arrest	1.27E‐29
GO:0010564	Regulation of cell cycle process	1.19E‐27
GO:0000082	G1/S transition of mitotic cell cycle	1.00E‐26
GO:0007346	Regulation of mitotic cell cycle	4.10E‐24
GO:0071156	Regulation of cell cycle arrest	5.40E‐23
GO:0000075	Cell cycle checkpoint	1.39E‐20
GO:2000602	Regulation of interphase of mitotic cell cycle	1.49E‐18
GO:2000045	Regulation of G1/S transition of mitotic cell cycle	1.49E‐16
GO:2000045	Regulation of G1/S transition of mitotic cell cycle	1.49E‐16
GO:0000084	S phase of mitotic cell cycle	4.75E‐13
GO:0071158	Positive regulation of cell cycle arrest	6.00E‐13
GO:0031571	Mitotic cell cycle G1/S transition DNA damage checkpoint	1.53E‐12
GO:0031575	Mitotic cell cycle G1/S transition checkpoint	2.71E‐12
GO:0090068	Positive regulation of cell cycle process	2.97E‐12
GO:0000086	G2/M transition of mitotic cell cycle	1.56E‐11
GO:0000087	M phase of mitotic cell cycle	4.06E‐10
GO:0045787	Positive regulation of cell cycle	9.05E‐08
Gene expression regulation‐related terms		
GO:0010467	Gene expression	8.82E‐71
GO:0006139	Nucleobase‐containing compound metabolic process	7.11E‐69
GO:0090304	Nucleic acid metabolic process	4.05E‐67
GO:0034641	Cellular nitrogen compound metabolic process	1.94E‐57
GO:0016070	RNA metabolic process	1.11E‐54
GO:0009059	Macromolecule biosynthetic process	1.93E‐44
GO:0034645	Cellular macromolecule biosynthetic process	1.97E‐44
GO:2000113	Negative regulation of cellular macromolecule biosynthetic process	5.72E‐41
GO:0010558	Negative regulation of macromolecule biosynthetic process	9.56E‐41
GO:0010629	Negative regulation of gene expression	1.54E‐40
GO:0032774	RNA biosynthetic process	9.47E‐40
GO:0010468	Regulation of gene expression	1.09E‐39
GO:0010628	Positive regulation of gene expression	4.79E‐39
GO:0051171	Regulation of nitrogen compound metabolic process	1.66E‐38
GO:0009891	Positive regulation of biosynthetic process	1.76E‐37
GO:0051254	Positive regulation of RNA metabolic process	4.72E‐37
GO:0045934	Negative regulation of nucleobase‐containing compound metabolic process	8.42E‐37
GO:0009890	Negative regulation of biosynthetic process	8.80E‐37
GO:0031328	Positive regulation of cellular biosynthetic process	1.68E‐36
GO:0010557	Positive regulation of macromolecule biosynthetic process	4.85E‐36
GO:0031327	Negative regulation of cellular biosynthetic process	8.05E‐36
GO:0051253	Negative regulation of RNA metabolic process	2.26E‐35
GO:0010556	Regulation of macromolecule biosynthetic process	2.72E‐35
GO:2000112	Regulation of cellular macromolecule biosynthetic process	5.58E‐35
GO:0045893	Positive regulation of transcription, DNA‐dependent	1.40E‐33
GO:0031326	Regulation of cellular biosynthetic process	2.14E‐33
GO:0045892	Negative regulation of transcription, DNA‐dependent	2.67E‐33
GO:0044249	Cellular biosynthetic process	2.93E‐32
GO:0051252	Regulation of RNA metabolic process	1.43E‐30
GO:2001141	Regulation of RNA biosynthetic process	1.91E‐29
GO:0006355	Regulation of transcription, DNA‐dependent	2.01E‐28
GO:0006351	Transcription, DNA‐dependent	1.86E‐27
GO:0019219	Regulation of nucleobase‐containing compound metabolic process	
GO:0006366	Transcription from RNA polymerase II promoter	
Cell adhesion‐related terms		
GO:0031589	Cell–substrate adhesion	3.19E‐6
GO:0007160	Cell–matrix adhesion	1.85E‐4
GO:0045785	Positive regulation of cell adhesion	9.38E‐4

### Subcellular layers of proteins in the PPI subnetwork

**Figure 6:**
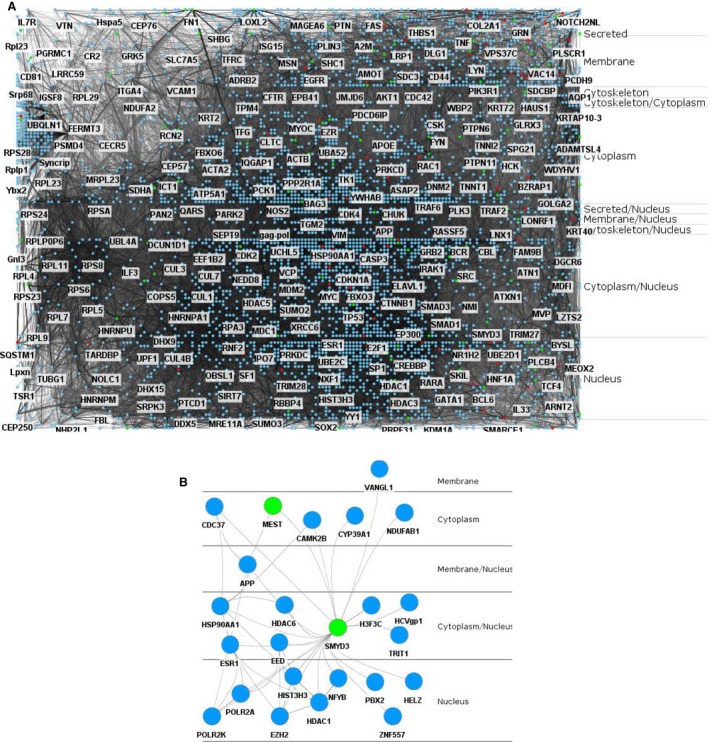
Subcellular layers illustrating the PPI network. (A) The complete DEG PPI network. (B) SMYD3‐central PPI subnetwork. Proteins were distributed according to their subcellular location without changing their interactions.

The proper subcellular localization of the proteins is extremely crucial because the appropriate location provides the physiological context for their functions, such as signal transduction, transcription regulation, protein modification, and complex formation. Cerebral program could array nodes in the PPI network into different subcellular layers maintaining their interactions. The total DEG PPI network was separated into 10 layers in the following percentages: secreted (6.5%), membrane (13.2%), cytoskeleton (0.5%), cytoskeleton/cytoplasm (0.3%), cytoplasm (26.4%), secreted/nucleus (0.8%), membrane/nucleus (0.3%), cytoskeleton/nucleus (0.45%), cytoplasm/nucleus (28.6%), and nucleus (23%; Fig. 6A). These results suggest the proteins in the total DEG PPI network distributed from extracellular to intracellular till nucleus.

There are currently 23 SMYD3‐interacting proteins reported and annotated in the PPI database. The most recognized SMYD3 function is histone methyltransferase, suggesting that this protein is mainly localized in the nucleus. To detect whether there were any possibilities for SMYD3 and its interacting proteins play roles in the nucleus, the proteins of the SMYD3‐central PPI network were also arrayed according to subcellular locations. Many SMYD3 interacting proteins, such as E2H2, HDAC1, NFYB, and POLR2A, were located in the nucleus, suggesting that SMYD3 might form functional protein complexes with these proteins to regulate gene expression (Fig. 6B). These results might reflect the intricate biological processes at the spatial level.

## Discussion

Globally, esophageal cancer is the eighth most common malignancy, as well as the sixth most common fatal cancer worldwide. Esophageal cancer has two histological types, adenocarcinoma and squamous cell carcinoma; the latter is among the four most common causes of death in China 29. More and more research has illustrated that systems biology approaches, such as network‐based methods, can be successfully applied to elucidate the molecular mechanisms of diseases 30, 31.

SMYD3 protein interacts with H3K4Me3‐modified histone tails, which facilitates its recruitment to the core promoter of transcriptionally active genes 32. To explore the potential roles or functions of SMYD3, a systems approach was applied by integrating public protein interaction data with DEGs resulting from SMYD3 knockdown to provide a full view. As shown in the three (downregulated, upregulated, and total DEGs) PPI subnetworks generated, thousands of proteins interact with the DEGs. This suggests that SMYD3 affects the expression of other proteins, and its knockdown impacts on cellular activities through the perturbation of the cellular protein network. Of the 23 SMYD3 directly interacting proteins, only the expression of MEST is downregulated. MEST is an imprinted gene with a hypermethylation promoter and is associated with cell invasion, as well as being a risk factor for cervical cancer and hepatocellular carcinoma 33, 34, 35. It is presumed that the knockdown of SMYD3 might impact on the expression of its directly interacting protein MEST.

The wide coverage of the Functional Annotation Chart provides a powerful tool to facilitate large‐scale gene function analysis from a network viewpoint. These functional terms are presumed to be significantly mediated by SMYD3 through its DEGs and could also be applied to explore the molecular roles of SMYD3 in ESCC tumor initiation and growth. The two terms of GO:0003682 of ‘chromatin binding’ and GO:0000979 of ‘RNA polymerase II core promoter sequence‐specific DNA binding’ have three repeated genes of GATA3, TP53, and YAP1 with fold changes of −2.71, −2.02, and −2.08, respectively, following knockdown of SMYD3 in ESCC. Yes‐associated protein 1, a key gene in the Hippo signaling pathway, is a crucial regulator pervasively activated in human malignancies 36. High levels of nuclear YAP1 are correlated with increased chromosome instability and aneuploidy in hepatocellular carcinoma 37. In breast cancer, Theodorou *et al*. found that GATA3 (GATA binding protein 3) is pivotal in mediating enhancer accessibility at regulatory regions involved in ESR1‐mediated transcription. GATA3 silencing results in a global redistribution of cofactors and active histone marks prior to estrogen stimulation. These results indicate that GATA3, when present on the chromatin, may serve as a licensing factor for estrogen–ESR1‐mediated interactions between *cis*‐regulatory elements 38. In this light, our data suggest that YAP1 and GATA3 are important target genes for future analysis on the impact of SMYD3‐mediated regulation of tumor‐associated genes.

To better understand the biological roles of the DEGs through the interactions with their protein partners, the total DEG PPI network was subjected to functional enrichment annotation by GO, which was also illustrated by a network. We show that the total DEG PPI network, perturbed by the knockdown of SMDY3, involves various biological activities, including the acknowledged and potential functions of SMDY3. Interestingly, the functional enrichment annotation map contains several cell motility‐related GO terms, for example, ‘cell–substrate adhesion’ and ‘cell–matrix adhesion’, indicating a function for SMYD3 in cancer cell metastasis. Direct evidence for SMDY3 participation in cancer cell metastasis has been found in our previous report from ESCC 14, as well as in bladder and colon cancer *in vitro* and *in vivo* 39, suggesting that SMYD3 is one of the key players stimulating migration and invasiveness of these cancer cells. On the other hand, SMYD3 is able to regulate cell signal transduction, cell cycle, and various biological effects, except the well‐known gene expression regulation, through a cascade of PPIs. The direct role for SMYD3 in the regulation of signal pathways has been reported, as SMYD3 mediates the methylation of MAP3K2 at lysine 260, which potentiates activation of the Ras/Raf/MEK/ERK signaling module 40. These results provide critical clues to explore the multiple functions of SMYD3 in the future.

Studies indicate that classical signaling pathways are composed of a series of genes or proteins, each linked by the order involved in signal transduction and response 41. We presume that the many canonical and noncanonical signals are also transduced by sequential protein interactions, arrayed in the proper layers. On the other hand, the roles or functions of the protein might vary according to its subcellular localization. For example, proteins located in the plasma membrane are primarily involved in cell adhesion, cytoskeleton, and cell signaling, whereas in the nucleus, proteins are mainly involved in transcription, ribosomal assembly, or chromatin remodeling 42. SMYD3 protein distributes both in cytoplasm and in nucleus or translocates from the cytoplasm into the nucleus, enabling its multiple functions in different subcellular localizations 43. Based on subcellular localization information, a pathway‐like view of total DEG PPI network was created, displaying the cellular locations of proteins and making it easier to understand the direction of information flow.

In summary, knockdown of SMYD3 causes the altered expression of its target genes, indicated by the PPI network, to directly or indirectly affect the signaling of extracellular membrane–cytoskeleton/cytoplasm–nucleus cascades, causing the altered expression of other DEGs, and consequently cause alterations in cell cycling, signal transduction, invasion, and metastasis.

## Data Accessibility

Both raw and treated expression data described in this study have been submitted to the NCBI GEO database (http://www.ncbi.nlm.nih.gov/geo/) and assigned the accession number GSE85419.

## Author contributions

XL and ZZ analyzed the data, interpreted the data, and wrote the manuscript. CC, SG, ZL, YL, YZ, HZ, JW, WX, and PZ analyzed the data and prepared the figures. LX, BW, and EL conceived and designed the study, and involved in supervision and funding acquisition. All the authors edited the manuscript prior to submission.

## Supporting information


**Appendix S1**. Functional Annotation Chart of differentially expressed genes following SMYD3 knockdown in ESCC.
